# High Glucose Level Impairs Human Mature Bone Marrow Adipocyte Function Through Increased ROS Production

**DOI:** 10.3389/fendo.2019.00607

**Published:** 2019-09-10

**Authors:** Tareck Rharass, Stéphanie Lucas

**Affiliations:** University of Littoral Côte d'Opale, University of Lille, CHU Lille, EA4490-PMOI-Physiopathologie des Maladies Osseuses Inflammatoires, Boulogne-sur-Mer, France

**Keywords:** adipocyte, bone marrow, mesenchymal skeletal stem cells, glucose, hyperglycemia, reactive oxygen species, oxidative stress, osteoporosis

## Abstract

Bone marrow adipocytes (BMAds) accumulate in aging, menopause, and metabolic diseases such as Type 2 diabetes. These osteoporotic conditions are associated with oxidative stress and hyperglycemia which are both considered as critical factors underlying bone fragility. Glucose excess and reactive oxygen species (ROS) are known to favor adipogenesis over osteoblastogenesis. In this study, we investigated whether high glucose exposure could determine dysfunction of mature BMAds, specifically through ROS production. The effects of low (LG, 5 mM) or high glucose (HG, 25 mM) concentrations were examined using human bone mesenchymal stromal cells (hBMSCs) in the time course of differentiation, and, up to 21 days once adipocytes were mature. HG did not alter the adipocyte differentiation process of hBMSCs. Yet, after 21 days under HG exposure, *PPARG, CEBPA*, and adiponectin mRNA expressions were decreased. These alterations were also observed following adipogenic inducer withdrawal as well as in adipocytes fully differentiated in LG then cultured in HG for the last 11 days. Without inducers, HG condition also led to decreased leptin mRNA level. Importantly, intracellular and extracellular ROS concentrations measured using Amplex Red were significantly raised by 50% under HG exposure. This rise was observed once adipocytes ended differentiation and was reproduced within the different cell culture settings without any cytotoxicity. Among genes involved in ROS metabolism, the mRNA level of the H_2_O_2_ generating enzyme NOX4 was found upregulated in the presence of HG. Following cell separation, mature BMAds were shown to overproduce ROS and to display the gene alterations in contrast to non-lipid-laden cells. Finally, a non-lethal treatment with a pro-oxidant agent under LG condition reduces the mRNA levels of *PPARG*, adiponectin, and leptin as the HG condition does in the absence of inducers, and amplifies the effect of glucose excess on gene expression. HG concentration drives mature BMAds toward altered expression of the main adipokines and transcriptional factors. These perturbations are associated with a rise in ROS generation likely mediated through enhanced expression of *NOX4*. Mature BMAds are thus responsive to changes in glucose and ROS concentrations, which is relevant regarding with their phenotype and function in age- or metabolic disease-related osteoporosis.

## Introduction

Over these last decades, Bone Marrow Adipocytes (BMAds) have been revealed as a new component interfering with bone homeostasis and the hematopoiesis function. The fat fraction within the bone marrow is well-known to accrue and to negatively correlate with bone density in aging (1) and postmenopausal (2) subjects. Moreover, the bone marrow fat fraction can increase in Type 2 diabetic patients (3–5) and is found closely associated with a poor glycemic control (5–9) which broadens its involvement in the compromised bone quality reported in such metabolic pathologies. Several *in vitro* studies and first *ex vivo* characterization of BMAds have emphasized how these specific adipocytes diverge from typical extramedullary adipocytes and release various products—adipokines, growth factors, inflammatory mediators, fatty acids—which can affect the differentiation, function, or survival of the bone-forming osteoblasts and/or the bone-resorbing osteoclasts (10). Furthermore, many local and systemic factors reciprocally regulate the commitment and differentiation of the bone marrow mesenchymal stem cells also referred to as Bone Mesenchymal Stromal Cells (BMSCs) toward either osteoblastogenesis or adipogenesis (11). Beyond these established characterizations, factors that regulate BMAd functions are barely studied.

Chronic hyperglycemia defines diabetes while severe delayed blood glucose clearance takes hold in aging and menopause. Over time, high glucose levels damage bone through biochemical modifications of protein matrix with accumulation of advanced glycosylation end-products (AGEs) (12) and cellular dysregulations such as reduced BMSC proliferation (13, 14). Besides, osteoblastogenesis has been shown to be diverted toward adipogenesis in human BMSCs (13–15), rodent bone marrow stromal cells (16, 17), osteoblast precursor (18), and osteoblast-cell lines (19, 20) upon high glucose concentration exposure. In this context, a higher production of Reactive Oxygen Species (ROS) has been reported (18) in line with ROS involvement in suppressing osteogenic signaling pathways while triggering those of adipogenesis (21). Moreover, hyperglycemia condition also interferes with mature osteoblast biomineralization (22) and with functions of fully differentiated extramedullary adipocytes. Indeed, for the 3T3-L1 adipocytes, the prototypical cell line to model adipogenesis and white adipocyte functions, differentiation, and maturation in high glucose concentrations attenuate the insulin-stimulated signaling pathway (23), increase the expression of several inflammatory and chemotactic mediators (23, 24), and elevate levels of mitochondrial and endoplasmic reticulum stress (25). Importantly, a significant rise in intracellular ROS production is induced in the presence of high glucose concentrations both in 3T3-L1 adipocytes (23, 26) and mature adipocytes isolated from visceral fat pads (27) and has been associated with adipocyte dysregulations in metabolic diseases. Glucose excess and ROS generation are thus closely intertwined in both adipogenesis and adipocyte dysfunction.

Finely-tuned and moderate ROS levels are required as messengers of redox-sensitive signaling pathways during adipocyte differentiation as evidenced for extramedullary adipocytes [reviewed in (28)] and BMAds (18, 29–31). Yet, a persistent and high production of ROS leads to oxidative stress with molecule oxidation contributing to insulin-resistance and altered adipokine secretion in white adipocytes (32). The ROS rate results from the combination of various generating enzymes and scavenging defense systems which are differently expressed in the course of stem cell commitment, differentiation, and maturation of adipocytes. ROS sources mainly include the mitochondrial respiratory chain and the Nicotinamide Adenine Dinucleotide Phosphate (NADPH) oxidases (28, 32). ROS levels are enhanced in extramedullary adipose tissues in both fat-depot- and age-related fashions (33) and in metabolic disorders (34). Remarkably, oxidative stress in bone stimulates resorption (35) while represses formation (36) and is considered as one of the key processes in the loss of bone integrity in aging (37) and post-menopausal state (38). Yet ROS production in BMAds has only been studied in differentiating BMSCs within the first 12 days (21, 29–31, 39) and the contribution and sensitivity of mature (fully differentiated) BMAds to a high oxidative status has never been assessed.

As exemplified (29–31, 39), BMAds derived from human BMSCs or rodent bone marrow stromal cells are commonly studied in DMEM containing a high glucose concentration (i.e., 4.5 g/L or 25 mM glucose). In this study, we thus addressed whether such high glucose (HG) condition may affect the function and ROS generation of human BMSC-derived BMAds once they have reached their mature state. Indeed, the impact of glucose excess in comparison to the normoglycemic or low glucose concentration (i.e., 1 g/L or 5.5 mM glucose) was reappraised during the first 10 days of differentiation and followed up to 21 days of culture. Moreover, the influence of a pro-oxidant agent was analyzed to determine if ROS generation is involved in HG-induced BMAd deregulation. Using various cell culture set-ups, gene expression analyses and direct H_2_O_2_ measurements in intracellular and extracellular compartments, our results sustain that HG concentration leads to a ROS overproduction that perturbs notably adipokine synthesis.

## Materials and Methods

### Cell Culture and Adipocyte Differentiation

Pre-screened human bone marrow mesenchymal stem cells now referred to as Bone Mesenchymal Stromal Cells (hBMSCs) were classically proliferated in high glucose (25 mM) DMEM medium, 10% fetal bovine serum (FBS), 1% L-glutamine and 1% penicillin/streptomycin (all from PanBiotech), up to 80% cell confluence to proceed with subsequent passages or adipocyte differentiation. All the experiments were performed between passages 7–9 for two donors (from Lonza; 19 year male, lot n°6F4393, and, 23 year female, lot n°423370).

hBMSC differentiation into BMAds was induced either in a low glucose (LG; 5 mM, to represent normal glycemia) or a high glucose (HG; 25 mM, to imitate severe diabetes) concentration. The two corresponding DMEM media (with FBS, L-glutamine, and penicillin/streptomycin) were supplemented with 1 μM insulin (PanBiotech), and other adipogenic inducers i.e., 0.5 μM dexamethasone (Dexa), 0.5 mM 3-isobutyl-1-methylxanthine (IBMX), and 50 μM indomethacin (Indo; all from Sigma). Adipogenic media were replaced every 3–4 days with fresh media up to 21 days of culture. Besides, in a third condition referred to as LG/HG, hBMSCs were differentiated in LG for the first 10 days (i.e., during the differentiation process) and in HG the following 11 days (i.e., once adipocytes are mature). Furthermore, to address the impact of adipogenic inducers [condition referred to as (+ind)], Dexa, IBMX and Indo were removed from media 2 days before the final analyzed time points (i.e., at day 12 or 19) and referred to as (–ind).

The cell differentiation and maturation were monitored using bright field microscopy up to 21 days; microscopic images were acquired and brightness/contrast adjustments were applied equally to every pixel in the images using Fiji/ImageJ (NIH).

### BMAd Treatments With Pro- and Antioxidant Reagents

Stock solutions of 10 mM tert-butyl hydroperoxide (TBHP) and 400 mM *N*-acetyl-*L*-cysteine (NAC) (all from Sigma-Aldrich) were freshly prepared in sterile distilled water and PBS, respectively. Fully differentiated BMAds (at day 13 or 20) were treated for 1 day with either 10 to 200 μM TBHP (pro-oxidant) or with 1–10 mM NAC (antioxidant).

### Separation of Non-lipid-laden and Lipid-Laden Cells

The procedure for separating lipid-laden and non-lipid-laden cell subpopulations was modified from Holt et al. (40). Differentiated cells were trypsinized on day 14 or 21 for a short duration (3–5 min) to allow detachment of non-lipid-laden cells only. The supernatant containing a majority of non-lipid-laden cells was collected and further processed with four centrifugation steps at room temperature (first, at 1,000 rpm for 5 min; second, at 2,000 rpm for 5 min; finally, two steps at 300 rpm for 10 min). At each step, the supernatant containing residual lipid-laden cells was carefully discarded, and the cell pellet corresponding to non-lipid-laden cells was resuspended in fresh media and vigorously mixed. Pelleted cells were either plated in the corresponding media for cell observation or used for subsequent analysis. After trypsin treatment, the remaining adherent cells, which mostly consist in lipid-laden cells i.e., BMAds, were washed twice with PBS and replenished with the corresponding media for 1 h before further processing.

The two cell subpopulations were lysed either in Extract-All (Eurobio) for mRNA expression analysis or lysis reagent (Life technologies) for ROS assays.

### Quantitative Real-Time Polymerase Chain Reaction

Total RNAs were extracted using Extract-All following the manufacturer's instructions. Residual contaminating genomic DNA was digested by 2.5 unit/μl of DNase I recombinant (Roche). Reverse transcription was performed from 1 μg to 100 ng (for separated cell subpopulations) RNA using Maxima first strand cDNA synthesis kit (Thermo Scientific).

Real-time PCR analysis was carried out with FastStart Essential DNA Green SYBR master mix using the LightCycler Nano Instrument (Roche). Primers were designed with OLIGO Primer Analysis Software 6 (Molecular Biology Insights) and used when efficiency was >1.8 as for the following gene sequences: *ADIPOQ* (Fwd 5′-AGC TCT GCC CGG TCA-3′, Rev 5′-GAT CTT GGT AAA GCG AAT GG-3′), *CAT* (Fwd 5′-AAA CCG CAC GCT ATG GCT G-3′, Rev 5′-TGG AGA ACC GAA CTG CGA TG-3′); *CEBPA* (Fwd 5′-ACT GGG ACC CTC AGC CTT G-3′, Rev 5′-TGG ACT GAT CGT GCT TCG TG-3′); *DGAT2* (Fwd 5′-GCC TCT TCT CCT CCG ACA C-3′, Rev 5′-CCG AAC TTG GTC TTG TGC TT-3′); *GLUT4* (Fwd 5′-ATG CTG CTG CCT CCT ATG AA-3′, Rev 5′-CAG TTG GTT GAG CGT CCC-3′); *GPX4* (Fwd 5′-GCC TTC CCG TGT AAC CAG T-3′, Rev 5′-GCG AAC TCT TTG ATC TCT TCG T-3′) (41); *LEP* (Fwd 5′-ATT TCA CAC ACG CAG TCA GT-3′, Rev 5′-GAA GAA GAT CCC GGA GGT-3′); *NOX4* (Fwd 5′-AGG ATC ACA GAA GGT TCC AAG C-3′, Rev 5′-TCC TCA TCT CGG TAT CTT GCT G-3′) (42); *PLIN2* (Fwd 5′-CAG AAG CTA GAG CCG CAA AT-3′, Rev 5′-ACG CCA CTG CTC ACG AG-3′); *PPARG* (Fwd 5′-GCT TCT GGA TTT CAC TAT GG-3′, Rev 5′-AAA CCT GAT GGC ATT ATG AG-3′); *RPL13A* (Fwd 5′-GCG GAT GAA CAC CAA CCC TT-3′, Rev 5′-GCA GCA TAC CTC GCA CGG-3′); *SOD2* (Fwd 5′-ACC TGC CCT ACG ACT ACG G-3′, Rev 5′-GGT ACT TCT CCT CGG TGA CG-3′). Amplifications were performed as follows: 10 min at 95°C for polymerase activation; 40 repeats of three-step cycling (20 s denaturation at 95°C, 10 s annealing at 54–61°C, 10–36 s elongation at 72°C) for amplification. A melting curve analysis confirmed product specificity. The relative mRNA expression of tested genes was calculated according to Ct values and primer efficiencies (E), then normalized to the ones calculated for the ribosomal protein L13a (*RPL13A*) housekeeping gene according to ${\text{E}}_{\text{gene}}^{-\text{Ctgene}}$/${\text{E}}_{\text{ref}}^{-\text{Ctref}}$. As mentioned, relative mRNA expression were appropriately expressed as fold level according to LG condition either at day 3 (d3) or d21 for each donor and data are presented as average values ± SEM.

### Cell Viability Assay

Cytotoxicity was assessed using CyQuant cell proliferation assay kit according to the manufacturer's instructions (Life technologies). hBMSCs were seeded on Corning Costar 96 well plates (Sigma-Aldrich), then differentiated and treated as aforementioned. The DNA-bound fluorescence was measured using Xenius XMA fluorescence microplate reader (Safas) at ~500 nm excitation and ~530 nm emission maxima in each lysed sample. The parameter settings (photomultiplier voltage of ~800–1,000 V; excitation duration of ~0.5 s; shaking duration of ~10 s prior measurement) were kept constant for all comparative set of experiments. Results were obtained from one single excitation in each sample well; yet, no photobleaching was detected after several repeated excitation steps on the same sample. A reference standard curve was created for converting sample fluorescence values into cell numbers. Each condition was assessed at least in triplicate. Data are presented as average values of cell amount ± SEM and expressed in % of cell viability compared to the initial cell number (i.e., at day 0).

### Reactive Oxygen Species Assay

Amplex Red hydrogen peroxide/peroxidase assay kit (Life technologies) was used to measure intracellular and extracellular reactive oxygen species (ROS) (29, 43, 44) following a procedure previously reported (45, 46). Cells seeded in Corning Costar 96 well plates were rinsed twice with PBS, then lysed with 50 μl/well of microplate lysis reagent (Life technologies) diluted to 4X in Krebs-Ringer phosphate buffer (KRPG, containing 145 mM NaCl, 5.7 mM Na_3_PO_4_, 4.86 mM KCl, 0.54 mM CaCl_2_, 1.22 mM MgSO_4_), during 10 min at 37°C, and mixed vigorously. Then, each cell lysate was mixed with a KRPG solution containing Amplex Red (AmR) dye (final concentration: 50 μM; DMSO < 0.1%) and horseradish peroxidase (HRP) enzyme (final concentration: 0.1 unit/ml), to a final volume of 200 μl/well, mixed thoroughly and incubated at room temperature for 10 min, in the dark. As for extracellular ROS levels, a volume of 50 μl of cell media (i.e., conditioning media, CM) was collected from each well, from an initial volume of 200 μl/well. Each CM sample were placed in new 96 well plates and thoroughly mixed with AmR/HRP solution as previously mentioned, to a final volume of 100 μl/well. Sample fluorescence was measured using Safas Xenius XMA spectrofluorimeter at maxima excitation and emission wavelengths of 570 and 585 nm, respectively. The parameter settings (photomultiplier voltage between 600 and 1,000 V; excitation duration of ~0.2 s; shaking duration of ~5 s prior measurement) were kept constant for all comparative set of experiments. Results were averaged from four excitations in each sample well, and excitations were performed at equal distance (0.5 mm distance deviation from the first excitation) in each well; neither photobleaching nor photo-induction (47) were induced after three repeated excitation steps on the same sample following our experimental procedure. A hydrogen peroxide (H_2_O_2_) standard curve was generated for converting sample fluorescence values into H_2_O_2_ concentrations. A stock solution of 20 μM H_2_O_2_ was freshly prepared in KRPG from 3% H_2_O_2_ solution (Sigma-Aldrich) to produce H_2_O_2_ concentrations ranging from 0 to 2 μM. Solutions were loaded with AmR/HRP mix and fluorescence intensities were measured as abovementioned. Each condition was examined at least in triplicate (i.e., 3–4 wells/condition). Data were calculated as average values of ROS concentrations in function of the cell number in the wells ± SEM and expressed either in nM/cell or in fold change compared to control.

### Statistical Analysis

Statistical analyses were performed using two-way Anova for kinetics or two-tailed unpaired Student's *t*-test with Prism 5 (GraphPad). *p* < 0.05 was considered significant. Data are presented as means ± standard error of the mean (i.e., SEM) from at least three independent experiments and two distinct donors.

## Results

### HG Level Does Not Modify hBMSC Differentiation Into Adipocytes but Alters BMAds Once Mature

We investigated whether glucose levels affect adipogenesis and/or maintenance of the functions of BMAds by cultivating hBMSCs in pro-adipogenic media [i.e., (+ind) with insulin] up to 21 days in the presence of either low glucose (LG, 5 mM) or high glucose (HG, 25 mM) concentration. As shown by light microscopy (Figure S1), hBMSC phenotype changes along the differentiation: cells slowly accumulate lipids up to 7 days of differentiation, and mainly display typical BMAd phenotype (i.e., cells rich in lipid droplets) from 14 days. In fact, the differentiated population does not only contain typical BMAds, but also a significant part of cells exhibiting a fibroblast-like phenotype i.e., non-lipid-laden cells as referred in Holt et al. (40). Of note, no major difference regarding cell morphology and proportions in any subpopulation is observed between the two glucose levels (Figure S1).

**Figure 1:**
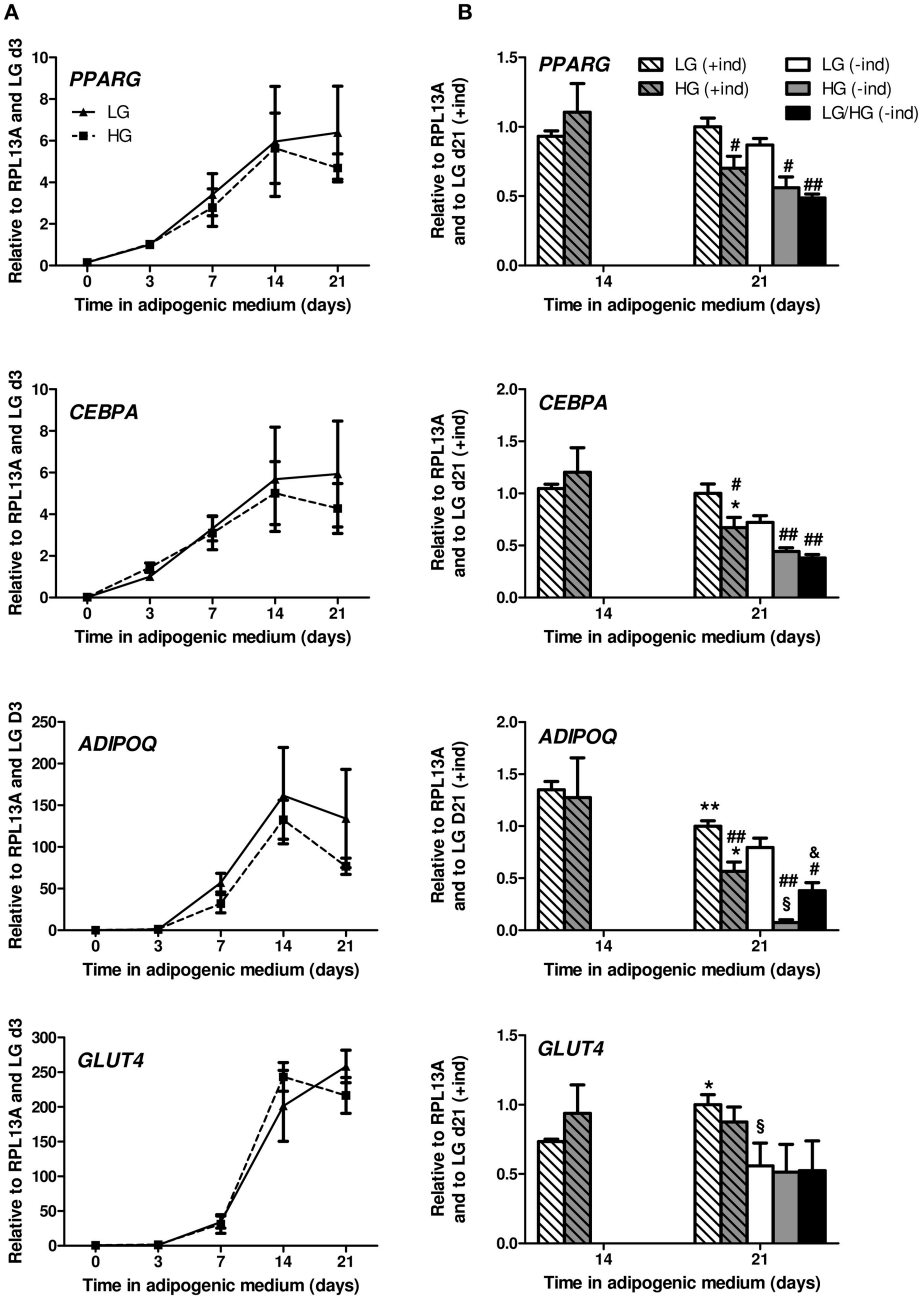
HG level does not impact adipocyte differentiation potential of hBMSCs but alters mature BMAds. **(A)** Kinetics of the expression of *PPARG, CEBPA, ADIPOQ*, and *GLUT4* genes observed after 0, 3, 7, 14, and 21 days in adipogenic differentiation medium under LG or HG concentrations. Data were obtained from one kinetic experiment performed in two donors and are expressed as relative mRNA fold changes according to values obtained at day 3 for each donor; data are presented as mean ± SEM. Using two-way ANOVA tests, the effect of time within adipogenic medium was found significant for all genes with *p* < 0.01. **(B)** Comparison of the mRNA levels measured after 14 and 21 days in differentiation media in the presence (+ind) or after the removal of the inducers 2 days prior to measurements (–ind), under LG and HG concentrations for 21 days or under HG condition for the last 11 days (LG/HG). Data were obtained from three independent experiments at day 14 and from six to seven independent experiments at day 21 with the two donors. Data are expressed as relative mRNA fold changes according to values obtained at day 21 in LG condition. Data are shown as mean ± SEM. ^*^p < 0.05, ^**^p < 0.01 compared to values obtained at day 14 for each glucose concentration; ^#^*p* < 0.05, ^##^*p* < 0.01 compared to values obtained in the respective LG condition. ^§^*p* < 0.05 compared to values obtained in the respective (+ind) condition. ^&^*p* < 0.05 compared to values obtained in the respective HG condition (using unpaired *t*-tests).

Following the mesenchymal stem cell commitment, the early differentiation step of adipogenesis is dependent on the expression of the two main transcriptional factors PPARG (peroxisome proliferator activated receptor gamma) and CEBPA (CCAAT/enhancer binding protein alpha) and requires around 7 to 10 days of stimulation by adipogenic inducers (15, 39, 40). The terminal differentiation step encompasses acquisition of typical adipocyte genes such as lipogenic factors (e.g., the main glucose transporter *GLUT4*, the diacylglycerol O-acyltransferase 2 enzyme *DGAT2* involved in fatty acid esterification into triglycerides) and adipokines (48), which leads to mature functional cells with larger lipid accumulation (39, 40, 48). To address if adipocyte differentiation was enhanced in HG conditions as previously reported (13–15), mRNA expression was analyzed at different days for the two donors (Figure 1A). As expected, the expression level of *PPARG* and *CEBPA* are significantly induced from day 3 (d3) to d14 in adipogenic media. Markers of the terminal differentiation such as the adipokines *ADIPOQ* (adiponectin; Figure 1A) and *LEP* (leptin; Figure S2A), *GLUT4* (Figure 1A) and *DGAT2* (Figure S2A) are expressed from d7 and markedly upregulated at d14. Yet, in line with microscopic observation, no modification can be observed between the LG and HG conditions supporting that in our settings, adipocyte differentiation is not modified in the presence of 25 mM glucose [Figure 1A and Figure S2A, compare (+ind)]. Finally from d14 to d21, the mRNA expression of the different markers was analyzed in several independent replicates for the two donors and appeared overall stabilized in the LG condition: *PPARG, CEBPA* (Figure 1B), and *LEP* (Figure S2A) are unchanged, though *ADIPOQ, GLUT4*, and *DGAT2* are significantly modified by −25, +36, and +61%, respectively; however such variations are clearly inferior to that observed from d7 to d14. Altogether, these data infer that at d21, culture-derived BMAds can be considered as mature.

While adipocyte differentiation was unaltered, some genes were significantly down-regulated in BMAds cultured in HG by d21 [Figure 1B, compare (+ind)]. Indeed, compared to d14, one supplementary week in HG leads to a decline in *PPARG* (*p* = 0.06), *CEBPA* and *ADIPOQ* mRNA levels. The expression level of these genes is also significantly decreased compared to LG at d21. As for *GLUT4, DGAT2*, and *LEP* (Figure 1B and Figure S2B) expression is not modified at d21 whatever the glucose concentration.

Adipogenic inducers (Dexa, Indo, IBMX) can be suspected to stimulate ROS production in adipocytes (49, 50). Consequently, we examined the impact of the removal of inducers (–ind) for the last 2 days [Figure 1B, see (+ind) vs. (–ind)]. The expression levels of *PPARG* and *CEBPA* tend to diminish; *ADIPOQ* mRNA level is affected in LG (−20%, *p* = 0.06) and more reduced in HG (−87%) upon inducer withdrawal. Such changes have already been observed in hBMSC-derived BMAds (40). Yet, the impact of glucose excess on the perturbed gene expression of *PPARG, CEBPA*, and *ADIPOQ* is preserved [Figure 1B, see LG (–ind) vs. HG (–ind)]. Of note, *LEP* mRNA (Figure S2B) is unchanged when inducers are removed in LG condition, but significantly decreased in HG condition.

One could argue that chronic exposure to HG level from the differentiation process has determined such alterations. We thus analyzed BMAds which were first differentiated in LG condition for 10 days, and, by then switched to the HG condition for the following 11 days [Figure 1B, see LG/HG (–ind) condition]. Strikingly, changes observed between LG and LG/HG conditions are similar to those reported above between LG and HG conditions, with reduced levels for *PPARG, CEBPA, ADIPOQ*, and *LEP*. The genes involved in lipogenesis such as *GLUT4* and *DGAT2* do not vary according to glucose concentration.

Our settings thus indicate that, upon a condition mimicking hyperglycemia, hBMSC-derived adipocytes functions are rapidly dysregulated with perturbed adipogenic transcriptional factors and adipokine levels.

### HG Level Enhances ROS Production Both Intracellularly and Extracellularly in BMAds Once Mature

**Figure 2:**
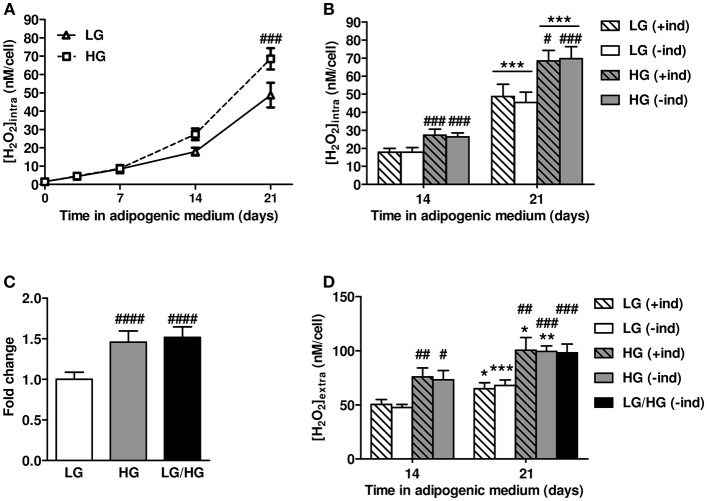
HG level stimulates intracellular and extracellular ROS production by mature BMAds. **(A)** Kinetics of intracellular reactive oxygen species (ROS) levels examined up to 21 days of BMAd differentiation and maturation under LG or HG concentrations in presence of adipogenic inducers (+ind). Data were obtained from six to nine independents measurements (at least three technical replicates for the two donors) and are expressed as intracellular H_2_O_2_ concentration in nM per cell, mean ± SEM. Using two-way ANOVA tests, the effect of time within adipogenic medium was found significant for both glucose concentrations with *p* < 0.0001; the effect of HG was found significant with *p* = 0.013 and Bonferroni post-test ^###^*p* < 0.001. **(B)** Comparison of intracellular ROS levels measured at days 14 and 21, under LG or HG conditions, and under continuous stimulation by inducers (+ind) or after their removal 2 days prior to measurements (–ind). Data were obtained from six to nine independents measurements and are expressed as abovementioned with mean ± SEM. ^***^p < 0.001 compared to values obtained at day 14 for the respective medium composition; ^#^*p* < 0.05, ^###^*p* < 0.001 compared to values obtained in the respective LG condition (using unpaired *t*-tests). **(C)** Intracellular ROS levels measured after 21 days in LG or HG concentrations or after 11 days in HG (LG/HG) following adipogenic inducer removal 2 days prior measurements (–ind). Data were obtained from eight to fourteen independents measurements, expressed as fold change compared to the LG condition, and are mean ± SEM. ^####^*p* < 0.0001 compared to LG condition (using unpaired *t*-tests). (**D**) Extracellular ROS levels assessed at days 14 and 21 in adipogenic medium, under LG, HG, or LG/HG conditions, with continuous stimulation by inducers (+ind) or after their removal 2 days before measurements (–ind). Data were obtained from four independents measurements from the two donors and are expressed as extracellular H_2_O_2_ concentration in nM per cell, mean ± SEM. ^*^p < 0.05, ^**^p < 0.01, ^***^p < 0.001 compared to values obtained at day 14 for the respective medium composition; ^#^*p* < 0.05, ^##^*p* < 0.01, ^###^p < 0.001 compared to values obtained in the respective LG condition (using unpaired *t*-tests).

Next, we addressed whether reactive oxygen species (ROS) metabolism is altered and involved in the impairment of BMAd functions by HG. Intracellular ROS levels were measured during the differentiation process (from d0 to d14) and the maturation phase (i.e., at d21) in hBMSC-derived BMAds using the Amplex Red (AmR) method (45–47). Intracellular ROS production doubles along the different time points up to d14 in adipogenic LG medium and accelerates while reaching the maturation step between d14 and d21 (Figure 2A). Importantly, ROS levels are similar whatever the glucose concentration up to d7 of differentiation, while they markedly rise in the presence of HG concentration from d14 (3-fold increase vs. HG d7) to d21. Consequently, and as also shown in Figure 2B, HG exposure raises up to 46% the ROS rate compared to LG at both days 14 and 21. To verify whether adipogenic inducers contribute in regulating ROS metabolism as suspected (49, 50), ROS levels were also measured at d14 and d21 after the removal of the inducers 2 days prior to measurements (Figure 2B). Interestingly, withdrawing inducers (–ind) did not affect the ROS production since levels remained comparable to those obtained following permanent inducer stimulation (+ind), under either LG or HG conditions. To emphasize that BMAds are able to produce more ROS in the presence of HG concentration once mature, we compared intracellular ROS levels in cells fully cultured in LG or HG conditions for 21 days with cells exposed to LG condition the first 10 days followed by 11 days under HG, i.e., LG/HG condition. BMAds derived from the LG/HG condition display a 52% higher ROS production than those under LG, and at a similar rate than BMAds generated in HG condition (Figure 2C).

Additionally, as adipocytes also produce extracellular ROS (51), we measured ROS levels 21 days after the induction of differentiation within conditioned media (Figure 2D). BMAds are found to profusely produce extracellular ROS as their levels exceed by 1.5- to 3-fold the intracellular ones for both glucose conditions (Figure 2B vs. Figure 2D). Yet, results recapitulate the aforementioned changes in intracellular ROS metabolism. First, BMAds exposed to HG condition release higher ROS levels (by ~55%) than cells under LG, which is in line with the stronger level of intracellular ROS metabolism found under the higher glucose concentration. Next, removing the inducers 2 days prior to measurements (–ind) do not influence on the extracellular ROS levels which remain similar to the ones measured under continuous stimulation by inducers (+ind). Finally, elevated extracellular ROS levels observed under HG do not result from any perturbations of the differentiation process since comparable results are found for BMAds under LG/HG condition (Figure 2D).

One could argue that the aforementioned changes in ROS levels may result from variations in cell amounts in response to ROS-mediated oxidative stress and underlying cell damages. We therefore measured the cell amounts by means of a fluorimetric assay based on cellular DNA content. We found that cell amounts are unmodified along the cell differentiation upon the different glucose conditions (Figure S3) confirming that ROS changes are independent from any alteration of cell viability. Additionally, since it has been reported that the use of fluorescent ROS sensors can have several pitfalls (45–47), we verified the specificity of the AmR probe for both intracellular and extracellular ROS levels as well as its sensitivity for ROS detection by spectrofluorimetry (Figure S4). Results show that the AmR method is reliable for detecting intracellular H_2_O_2_ following cell lysis (Figure S4A) and for measuring extracellularly released H_2_O_2_ levels without any reaction from medium compounds (Figure S4B) or photo-induction of the probe (Figure S4C). Taken together, these data substantiate that changes in ROS levels found in Figure 2 do not result from any artifact but rather depend on glucose conditions.

### Glucose Modulates ROS Metabolism of Mature BMAds in a NOX4-Dependent Manner

We then aimed to identify which intracellular enzymatic system mediates the rise in ROS metabolism under HG condition. Among multiple enzymatic systems, mitochondria are well-known for being the main source for intracellular ROS production. We thus evaluated the mRNA expression levels of *SOD2* (superoxide dismutase 2), a mitochondrial enzyme converting the superoxide anion into H_2_O_2_ and whose activity is tightly associated with the function of the mitochondrial electron transport chain that leaks the superoxide anion (52). As for the antioxidant defense system, we focused on the expression of *CAT* (catalase) and *GPX4* (glutathione peroxidase 4) which both scavenge excessive amounts of H_2_O_2_ and have already been found to be well-expressed in hBMSC-derived adipocytes (39) and BMAds from aging mice (53), respectively.

**Figure 3:**
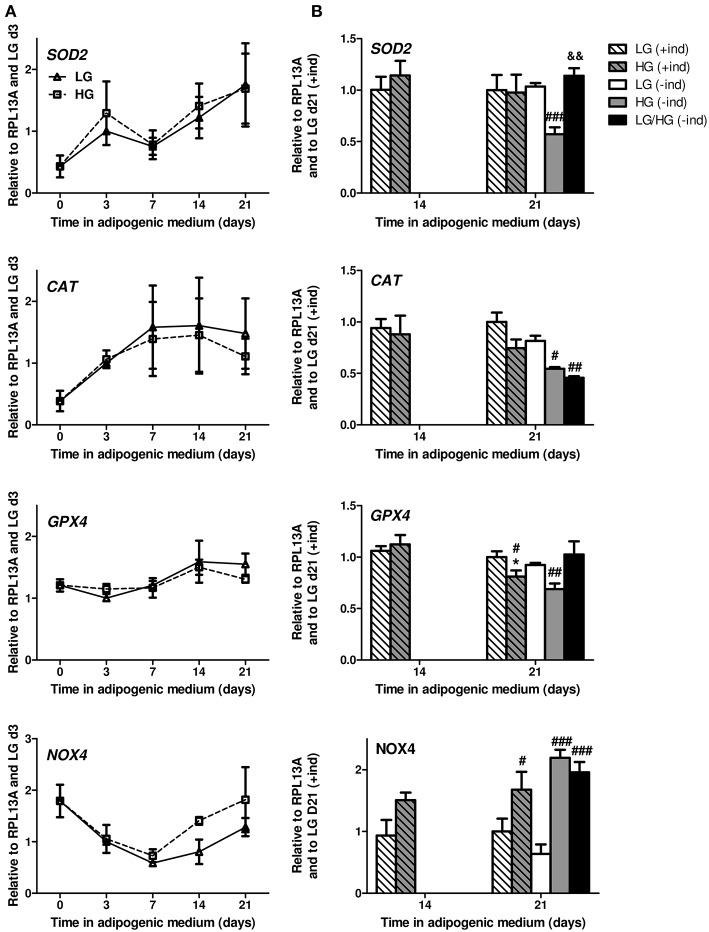
ROS are mainly produced in a NOX4-dependent manner in mature BMAds. **(A)** Kinetics of *SOD2, CAT, GPX4*, and *NOX4* mRNA levels observed after 0, 3, 7, 14, and 21 days in adipogenic differentiation medium under LG or HG concentrations. Data were obtained from one kinetic experiment performed in two donors and are expressed as relative mRNA fold changes according to values obtained at day 3 for each donor; data are presented as mean ± SEM. Using two-way ANOVA tests, the effect of time within adipogenic medium was found significant for all genes with *p* < 0.02. **(B)** Comparison of the mRNA levels measured after 14 and 21 days in differentiation media in the presence (+ind) or after the removal of the inducers 2 days prior to measurements (–ind), under LG and HG concentrations for 21 days or under HG condition for the last 10 days (LG/HG). Data were obtained from three independent experiments at day 14 and from six to seven independent experiments at day 21 with the two donors. Data are expressed as relative mRNA fold changes according to values obtained at day 21 in LG condition. Data are shown as mean ± SEM. ^*^p < 0.05, compared to values obtained at day 14 for each glucose concentration; ^#^*p* < 0.05, ^##^*p* < 0.01, ^###^*p* < 0.001 compared to values obtained in the respective LG condition. ^&&^*p* < 0.01 compared to values obtained in the respective HG condition (using unpaired *t*-tests).

Regardless of the glucose level, *SOD2* and *CAT* mRNA levels rapidly increase during adipocyte differentiation (Figure 3A) as already reported (39). The expression level of *GPX4* is stable during the first week of differentiation and slightly progresses from d7 to d14. In LG condition, all genes reach their maximal expression by d14 (Figure 3B). Yet HG exposure for 21 days leads to a decrease in *GPX4* and *CAT* (*p* = 0.06) levels compared to LG condition, with no change for *SOD2* (Figure 3B). The removal of inducers does not disrupt the expression of these genes for BMAds in LG or HG medium [Figure 3B, see (+ind) vs. (–ind)]. Nevertheless, the absence of inducers reveals better the impact of HG concentration on the expression of *CAT, GPX4*, and *SOD2* which are down-regulated compared to LG condition. Surprisingly, a 11 day exposure to HG concentration (LG/HG condition) does not totally reproduce the impact of cell exposure in the high glucose concentration from the differentiation process: indeed, *SOD2* and *GPX4* levels are unchanged compared to LG and increased compared to HG condition. Of note, the expression of *CAT* mRNA remains significantly diminished in LG/HG compared to LG (Figure 3B). Altogether, these data do not support any primary involvement of these systems in the rise of ROS generation under HG exposure (either HG or LG/HG) as observed (Figures 2B,D): discrepancies are obvious between the gene expression pattern of the H_2_O_2_ generating enzyme *SOD2* and ROS amounts in our different conditions. As for the *CAT* and *GPX4* gene expression, the HG-induced decrease could be suspected to contribute to ROS accumulation after 21 days but not from 14 days as observed (Figure 2B).

Consequently, we investigated whether HG-mediated ROS metabolism involves NADPH oxidases (NOX). These membrane-bound enzyme complexes lead to both intracellular and extracellular ROS (i.e., H_2_O_2_) production (51, 54). NOX4 is reported to be the major isoform in extramedullary adipocytes and to be critically regulated during both the differentiation process (21, 51) and the exposure to nutrients excess (55) of 3T3-L1 adipocytes. *NOX4* mRNA expression drastically decreases during the early differentiation stage (up to d7, −67% compared to d0) in a comparable manner between LG and HG levels (Figure 3A), which reproduces previous results for the differentiating 3T3-L1 cell line (51). Since ROS levels increase during such time period (Figure 2A), it is therefore unlikely that NOX4 constitutes the main source for ROS production during the differentiation process. Interestingly, from d7 to d21, *NOX4* mRNA levels increase with LG and HG exposure, respectively, leading to 72 to 100% of the expression level at d0 (Figure 3A). Indeed, compared to LG, *NOX4* mRNA level is up-regulated in the HG condition by 61% (*p* = 0.11) and 68% at d14 and d21, respectively (Figure 3B). Besides, the absence of inducers does not significantly affect *NOX4* expression; yet, the impact of glucose concentration is more emphasized with a ~3-fold increase of *NOX4* levels in HG and LG/HG conditions compared to the LG one [Figure 3B, see (+ind) vs. (–ind)]. From d14 to d21 and in the different culture conditions, ROS generation and *NOX4* expression evolve according to a comparable pattern, which supports the central involvement of the enzyme in the glucose-induced ROS production of mature BMAds.

### Glucose-Induced Stimulation of ROS Metabolism Is Restricted to Mature Lipid-Laden BMAds

**Figure 4:**
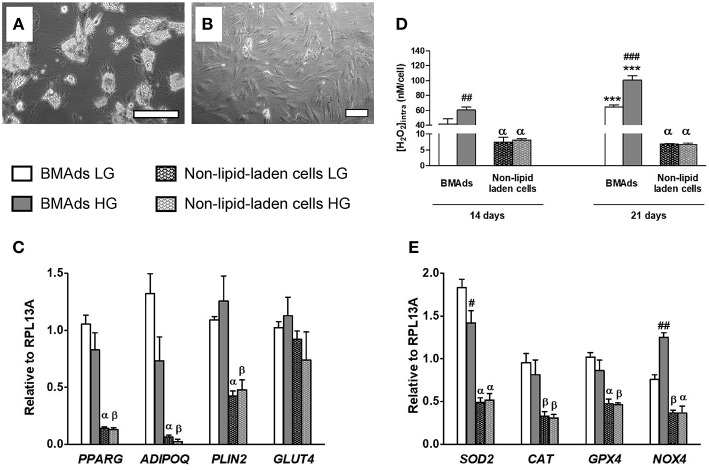
HG level stimulates ROS production specifically in the lipid-laden BMAds subpopulation. **(A,B)** Microscopic images showing the two different cell populations which were separated as described in Materials and Methods. Adherent cells remaining after trypsin treatment are mostly lipid-laden BMAds as shown in **(A)** after 1 h of recovery in the corresponding adipogenic medium. The detached cell subpopulation was also seeded in the corresponding adipogenic medium to assess cell purity; as shown in **(B)**, most cells are non-lipid-laden cells. Scale, 50 μm. **(C)** The mRNA expression level of adipocyte genes (*PPARG, ADIPOQ, PLIN2, GLUT4*) were measured in the lipid-laden BMAd-enriched subpopulation and in the non-lipid-laden cell-enriched subpopulation to prove cell separation efficiency. Gene expression analysis was performed after 21 days in LG or HG conditions; inducers were removed 2 days before cell separation. Results are Mean ± SEM from four independent experiments (two technical replicates with the two donors). **(D)** Intracellular ROS levels were measured in both cell subpopulations following their separation. Measurements were performed after 14 or 21 days in LG or HG conditions and after removal of the inducers for the last 2 days. Intracellular H_2_O_2_ concentration data (expressed in nM per cell) are Mean ± SEM from three independent experiments (technical replicates with the two donors). **(E)** The mRNA expression level of several genes involved in ROS metabolism (*SOD2, CAT, GPX4*, and *NOX4*) were measured in the lipid-laden BMAd-enriched subpopulation and in the non-lipid-laden cell-enriched subpopulation to analyse their respective contribution. Results are Mean ± SEM from four independent experiments [as described in **(C)**]. ^***^p < 0.001 vs. lipid-laden BMAds after 14 days in adipogenic medium at the corresponding glucose concentration; ^β^*p* < 0.05, ^α^*p* < 0.001 vs. lipid-laden BMAds at the corresponding glucose concentration and time analysis; ^##^*p* < 0.01, ^###^*p* < 0.001 between the two glucose concentrations (using unpaired *t*-tests).

As shown in Figure 3A and in previous works (21), NOX4 is highly expressed in adipocyte precursors. Yet, as commonly observed, the hBMSC-derived fully differentiated cell population displays both lipid-laden (i.e., mature BMAds) and non-lipid-laden cells which are barely studied (Figure S1). To characterize how each subpopulation contributes to the ROS production, we thus performed an isolation procedure to get enriched fractions in lipid-laden and non-lipid-laden cells after 21 days in LG or HG adipogenic media. As shown in Figures 4A,B, each cell fraction is remarkably enriched in each specific cell type. Gene expression analysis demonstrates that the lipid-laden cells considerably express higher *PPARG* (>6-fold), *CEBPA* [(>19-fold), not shown] and *ADIPOQ* (>21-fold) mRNA levels than non-lipid-laden cells (Figure 4C). Besides HG exposure results in decreased expression levels of *PPARG* (*p* = 0.15), *CEBPA* (*p* = 0.07), and *ADIPOQ* (*p* = 0.06) in the lipid-laden subpopulation, as shown for the whole cell populations in Figure 1B. Of note non-lipid-laden cells express *PLIN2* (perilipin2) which encodes for proteins recovering nascent lipid droplets during adipocyte differentiation (56). Yet, *PLIN2* mRNA level remains 2.6-fold lower expressed in non-lipid-laden cells than in lipid-laden cells. *PLIN1* (perilipin 1), the most specific and abundant protein covering mature triglyceride droplets in adipocytes (56), is drastically reduced by 50-fold in the non-lipid-laden subpopulation compared to the lipid-laden one (data not shown). Interestingly, *GLUT4* mRNA expression was found similarly expressed in the two cell subpopulations. These data thus confirm the cell separation efficiency and suggest that non-lipid-laden cells can be considered as immature cells committed in the adipocyte lineage that, yet, already rely on glucose metabolism.

We then quantified intracellular ROS concentrations in each subpopulation (Figure 4D). Regardless of glucose level, lipid-laden cells produce much higher ROS levels that non-lipid-laden cells, confirming that BMAds constitute the main cell source for ROS production in our model. Interestingly, changes in glucose levels did not affect the ROS levels in non-lipid-laden cells. In contrast, HG condition enhances ROS generation in the fully functional lipid-laden cells by about 50% compared with LG, both at d14 and d21, as found for the whole cell populations (Figure 2B). These findings support that the up-regulation of ROS metabolism by increased glucose level is connected to a molecular machinery which takes place only once cells were fully differentiated.

Expressions of genes controlling the ROS rate were also studied within each cell fraction (Figure 4E). The H_2_O_2_ generating enzyme *SOD2* is found 3-fold better expressed in BMAds than in non-lipid-laden cells and is down-regulated by 23% upon HG exposure, in a similar way to the whole cell population (Figure 3B). Importantly, BMAd subpopulation displays the highest levels of the other H_2_O_2_ generating enzyme *NOX4* which, furthermore, is up-regulated by 65% in the presence of HG concentration (Figure 4E), confirming that the increased expression induced by HG exposure is due to mature BMAds within the whole cell population. Regarding the antioxidant enzymes, *CAT* and *GPX4* mRNA levels are ~2-fold less abundant within non-lipid-laden cells compared to BMAds, yet without any significant impact of the glucose concentration. Altogether, our data support that the glucose-induced enhancement of ROS generation is restricted to mature BMAds and most likely relies on the activity of NOX4.

### Impairment of BMAd Function Under HG Level Is Mediated Through Enhanced ROS Metabolism

**Figure 5:**
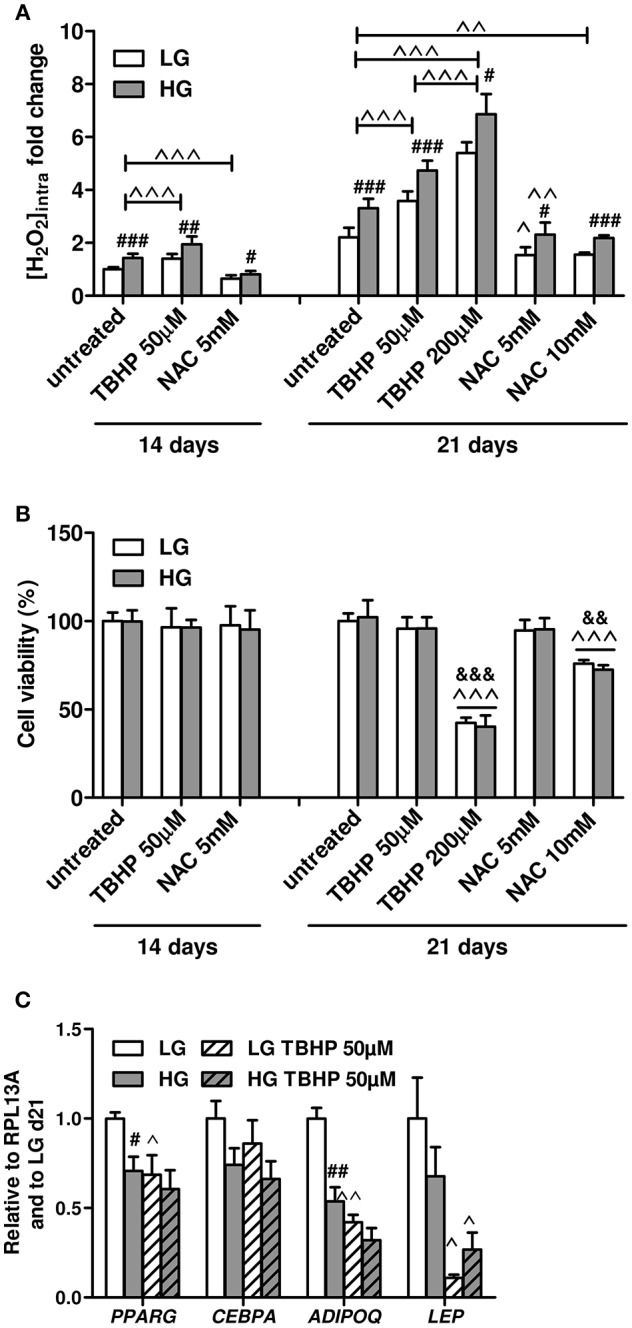
Low doses of TBHP enhance ROS production in mature BMAds in a non-lethal manner and impair the gene expression of typical adipocyte genes. **(A)** Effects of 1 day treatment of mature BMAds after 14 or 21 days in LG or HG conditions with TBHP (50 and 200 μM) or NAC (5 or 10 mM) on intracellular ROS levels. Inducers have been removed from cell culture media 2 days prior to measurements. Data were obtained from six to ten independent experiments (at least three technical replicates with the two donors) and expressed as relative fold changes of measured H_2_O_2_ concentration according to values obtained at d14 in LG condition. Data are shown as mean ± SEM. Significant elevations of ROS levels between d14 and d21 were found but are not indicated. ^###^*p* < 0.001, ^##^*p* < 0.01, ^#^*p* < 0.05 between the two glucose concentrations for the respective treatment. ^∧∧∧^*p* < 0.001, ^∧∧^*p* < 0.01, ^∧^*p* < 0.05 compared to untreated conditions for the respective glucose concentration (using unpaired *t*-tests). **(B)** Cell viability measured at d14 and d21 in untreated BMAds and following treatments with different doses of TBHP or NAC, under LG or HG conditions after removal of the inducers. Data were obtained from four independent experiments (two technical replicates with the two donors) and are presented as percentage of cell viability ± SEM compared to the cell amount at d0. ^∧∧∧^*p* < 0.001 compared to untreated conditions for the respective glucose concentration; ^&&&^*p* < 0.001, ^&&^*p* < 0.01 compared to the lowest dose for the respective pharmacological agent (using unpaired *t*-tests). **(C)** Effects of a 1 day treatment with TBHP (50 μM) of mature BMAds at d21, in LG or HG conditions, on the expression levels of *PPARG, CEBPA, ADIPOQ*, and *LEP*. Inducers have been removed 2 days prior to measurements. Data were obtained from three to six independent experiments including the two donors. Data are expressed as relative mRNA fold changes according to values obtained at d21 in LG condition, and are mean ± SEM. ^#^*p* < 0.05, ^##^*p* < 0.01 compared to values obtained in the respective LG condition; ^∧∧^*p* < 0.01, ^∧^*p* < 0.05 compared to untreated conditions for the respective glucose concentration (using unpaired *t*-tests).

Next, we investigated whether the ROS excess produced during the HG concentration exposure could mediate the variations in the expression of the key adipogenic transcriptional factors and adipokines of BMAds observed at d21. We first assessed the responsiveness of the cells to treatments that artificially modulate intracellular ROS levels. BMAds were treated with different doses of pro- or antioxidant reagents, i.e., TBHP or NAC, respectively, for 1 day in the absence of inducers, under either LG of HG concentrations, prior to measurements of ROS levels, and cell viability (Figure 5). At both d14 and d21, exposing BMAds in LG to 50 μM TBHP increases the ROS levels to the ones observed for cells under HG alone, while 5 mM NAC in HG decreases the ROS rate to that of LG alone (Figure 5A). Besides, more ROS amount can be accumulated in BMAds treated with 50 μM TBHP and without any change in cell viability (Figure 5B). In contrast, the highest dose of TBHP (200 μM) triggers oxidative stress since the further rise in ROS levels is associated with a 60% loss of cells. Using NAC at either 5 or 10 mM lowers at a rather similar degree the intracellular ROS concentration at d21. Yet treatment with the highest NAC concentration results in cytotoxicity. Furthermore, in contrast to TBHP treatment, one has to point out that analyses of gene expression following NAC treatment do not appear advisable since either 5 or 10 mM of the reagent lead to a marked remodeling of lipid droplets (not shown) as already reported for adipocytes differentiated from a bone marrow stromal cell line (57), suggesting side effects in addition to the antioxidant activities. Overall, these data indicate that mature BMAds are well-responsive to any modulation of the redox state and handle higher ROS levels whatever the glucose concentration.

We therefore examined gene expression in mature BMAds under LG or HG conditions in the presence of a non-lethal dose of the pro-oxidant TBHP (50 μM; Figure 5C). Interestingly, TBHP treatment in LG condition results in similar decreases of *PPARG* and *ADIPOQ* mRNA levels than those observed in the HG condition alone. While *LEP* mRNA level tends to be lower in HG condition compared to the LG one, its expression is remarkably reduced in the LG TBHP condition compared to LG alone. Indeed, the transcriptional regulation of *ADIPOQ* and *LEP* genes display a high sensitivity to an increase in ROS amounts since both mRNA levels are further decreased in HG TBHP condition compared to HG alone (−40%, *p* = 0.06 and −60%, respectively). Of note, *CEBPA* expression levels tend to be reduced in HG condition (*p* = 0.09) but are unmodified in the presence of TBHP whatever the glucose concentrations, which suggests that the HG-mediated down-regulation in *CEBPA* mRNA level most likely relies on ROS-independent mechanisms. Altogether, the TBHP-induced increase in ROS levels mimics the down-regulation in the gene expression of *PPARG* and *ADIPOQ* observed in the HG condition (Figure 1B) and exacerbates the reported effect of HG on *LEP* expression (Figure S2), sustaining that HG-induced mature BMAd dysfunction is mediated by augmented intracellular ROS formation.

## Discussion

The involvement of various signaling pathways and mediators such as ROS has been substantially investigated in the BMAd differentiation process (11, 21). Yet, factors which regulate mature BMAd function are still poorly characterized while these specific adipocytes are considered as active cells interacting within the bone and bone marrow microenvironment (10, 11). Our study reports for the first time that mature BMAds exposed to a HG concentration display several alterations in the expression of the main adipokines and transcriptional factors involved in the maintenance of adipocyte function. These perturbations are associated with a rise in ROS generation likely mediated through the enhanced expression of *NOX4*. Actually, a non-lethal treatment with a pro-oxidant agent under LG condition reduces the mRNA levels of *PPARG* and *ADIPOQ* as the HG condition does, and amplifies the effect of glucose excess on *LEP* expression. Using various cell culture set-ups, several findings sustain that HG can regulate mature BMAd function. Firstly, examinations of gene expression, cell amount and ROS metabolism in the time course of adipocyte differentiation did not reveal any impact of glucose concentration. Secondly, any potential conditioning from the differentiation step has been analyzed and then discarded by using BMAds once fully differentiated under LG concentration. Indeed, an 11 day exposure to HG of these BMAds (LG/HG condition) recapitulates the modifications observed when cells were cultivated from differentiation in HG medium. Besides, removing adipogenic inducers once cells are differentiated does not suppress the ROS production and the gene expression profile induced by glucose. Finally, mature BMAds isolated from non-lipid-laden cells were found being the main cell source responsible of ROS production as well as being modulated by glucose with regard to gene expression alterations and ROS metabolism.

Mature BMAds are thus responsive to changes in glucose and ROS concentrations which is highly relevant regarding their phenotype and functionality in age- or metabolic disease-related bone disorders. Compared to extramedullary adipocytes, mature BMAds are characterized by low expression levels of *Pparg, Adipoq*, and *Lep* which are further reduced with the aging of mice (53). BMAds from Type 2 diabetic patients display a marked decrease in ADIPOQ expression and secretion with unchanged *PPARG* expression, and higher LEP mRNA and protein levels compared to BMAds from non-diabetic patients (4). Here we demonstrated that either HG-triggered ROS generation or artificially-induced ROS excess can alter *PPARG* and *ADIPOQ* expression without inducing any cytotoxicity. Interestingly, *PPARG* and *ADIPOQ* mRNA expressions have been shown to be suppressed by H_2_O_2_ in a dose-dependent manner in 3T3-L1 adipocytes (34). PPARG expression level and activity in mature extramedullary adipocytes is considered crucial to maintain adiponectin and other lipogenic enzymes expression level (58) and to repress inflammation (59) resulting in insulin-sensibility preservation (58). In our study, TBHP treatment in LG concentration reproduced the diminished expression levels of *PPARG* and *ADIPOQ* observed in HG condition (Figure 5C), supporting that the glucose-induced elevation in ROS production contributes to the regulation of their expression. Besides, in contrast to *PPARG, ADIPOQ* expression—whose mRNA level is further reduced in the presence of the pro-oxidant agent in HG condition—appears highly sensitive to the ROS rate in mature BMAds. This is a reminiscent feature of white adipocytes as reported through previous relationships between oxidative stress and ADIPOQ level in the 3T3-L1 model, in the visceral fat pads of mouse models and in human plasmas (34). Regarding the regulation of *LEP* expression in BMAds, several factors are likely involved. The impact of HG concentration and ROS metabolism is only revealed once inducers are removed from media (Figure S2 and Figure 5C). Actually, Dexa—one of the adipogenic inducers—has already been shown to stimulate the expression and release of LEP in hBMSC-derived adipocytes (60) and human primary cultivated BMAds (61). It is thus likely that, in our context, the impact of Dexa outcompetes the glucose-triggered effect when inducers are present. Interestingly, in the absence of Dexa, a pro-oxidant environment results in a tremendous decrease in *LEP* mRNA levels both in LG and HG conditions (Figure 5C). Altogether our data highlight that in a context of increased oxidative stress, such as the one triggered in aging (37), BMAds display an altered phenotype with a compromised capacity to synthetize ADIPOQ and LEP as already reported for mice (53) and humans (62). Such pro-oxidant status could also underlie the defect in *ADIPOQ* expression observed in the BMAds from Type 2 diabetic patients (4) while other factors predominate in the control of *LEP* expression.

Our study also provides evidence that the negative impact of hyperglycemia on bone is connected to an enhanced extracellular ROS production by mature BMAds, as supported with extracellular ROS concentrations higher than intracellular ones (Figure 2). Comparable amounts of H_2_O_2_ severely decrease viability of human osteoblast-like cells (63, 64). An excessive oxidative stress also promotes osteocyte apoptosis which, given the critical role of these main bone cells in bone remodeling, results in an increased and unbalanced bone resorption (65, 66). Conversely, counteracting oxidative stress improves osteocyte viability and cytokine expression (67), and prevents osteoblast apoptosis in aged (68) or gonadectomized (37) mice. Besides, differentiating osteoblasts from hBMSCs express less antioxidant enzymes than differentiating adipocytes (39). As previously emphasized, increased ROS metabolism also represses osteoblastogenesis (17, 69) and promotes adipogenesis (21, 31). Beyond these cell processes, oxidative stress combined with hyperglycemia accelerate AGEs formation and accumulation, such as pentosidine which is more and more considered as a critical determinant in the BMD-independent bone frailty of metabolic diseases (70–72). Our findings thus expand the deleterious roles of BMAds in Type 2 diabetes skeletal defects: besides compromising osteoblastogenesis, BMAds could further damage bone quality and properties by releasing more ROS in the context of uncontrolled hyperglycemia leading altogether to an exacerbation in AGEs formation.

HG-triggered ROS elevation in mature BMAds is likely associated with an increased NOX4 activity. The transcriptional regulation of *NOX4* has been shown to give account on its enzymatic activity in cell culture (51, 73). Among the different NOX isoforms, NOX4 directly and constitutively produces H_2_O_2_ (54), and, has been reported to be the main isoform expressed in adipose tissues (51). In our study, both a rise in ROS amounts and *NOX4* expression levels are observed in BMAds exposed to HG concentration, either from the maturation step (i.e., from d14, Figures 2A, 3A), in the presence or absence of adipogenic inducers or after a shorter exposure time to HG (LG/HG condition) (Figures 2B, 3B). In contrast, *SOD2* mRNA levels are found either unchanged or even decreased upon inducer withdrawal when BMAds are cultured in HG concentration (Figure 3B). The expression levels of enzymes involved in ROS detoxification—such as CAT and GPX4—are also down-regulated in HG condition, which could contribute to a higher ROS rate. Yet, such regulation is at most observed while adipogenic inducers are absent and within a moderate range [e.g., −34% for *CAT*, LG (–ind) vs. HG (–ind)] compared to the one observed for NOX4 (+245%, LG (–ind) vs. HG (–ind), Figure 3B). Moreover, the gene expression pattern of *NOX4* in our experiments is consistent with data from the 3T3-L1 preadipocyte cell line: while adipocyte differentiation is associated with decreased expression of NOX4 (51), fully differentiated adipocytes cultured in the presence of 25 mM glucose display a higher ROS generation which is concomitant with increased NOX4 activity (55). Indeed in that model, glucose excess was shown to be metabolized through the pentose phosphate pathway to provide larger amounts of NADPH which are cofactors of NOX enzymes. Besides, HG condition stimulates NOX4 translocation to lipid rafts of the plasma membrane (55). Though these findings have not been confirmed in hBMSC-derived adipocytes, it has to be pointed out that NOX4 activity is considered to provide continuously and independently of any activator H_2_O_2_ which is a membrane-diffusible and stable ROS (54). The HG-induced elevation in ROS levels observed both extracellularly and intracellularly is therefore likely ascribed to the increased *NOX4* expression in mature BMAds.

Finally, hBMSCs or rodent bone marrow stromal cells are usually grown, differentiated and matured into different cell lineages in DMEM containing a HG concentration, i.e., with 25 mM glucose (29–31, 39). Such cell culture practice is also used for the 3T3-L1 cell line despite the induction of several adipocyte alterations (24, 26, 55) as emphasized above. The requirement of such HG concentration has been recently reappraised in the different handling steps of the 3T3-L1 cell line: a 3 day pulse with 25 mM glucose was demonstrated to be needed during the differentiation process and not the proliferation, nor the maturation of the cells (74). Here, our study provides evidence that hBMSC differentiation into adipocytes is unaltered when using a LG concentration. Besides, maturing adipocytes in the presence of HG concentration results in several functional alterations with decreased *PPARG* and *ADIPOQ* levels, and increased ROS metabolism. With an increasing interest for BMAds in energy and nutrient metabolism perturbations, a particular attention regarding glucose concentration in culture conditions should thus be brought to better decipher BMAd involvement and regulation using *in vitro* models.

In conclusion, beyond being accrued in Type 2 diabetes (3–5), mature BMAds release higher ROS levels in their microenvironment in response to HG concentration exposure. This ROS overproduction induces in mature BMAds defects in *PPARG* and *ADIPOQ* expressions without any cytotoxicity. Considering that excessive ROS production is closely associated with an inflammatory response and impaired insulin signaling in extramedullary adipocytes, further dysfunctions in BMAds in the settings of hyperglycemia could be expected and are worth investigating.

## Data Availability

The datasets generated for this study are available on request to the corresponding author.

## Author Contributions

TR and SL designed the study, performed cell culture, qPCR and microscopy, analyzed the data, and wrote the manuscript. TR carried out microfluorimetry experiments. SL supervised the study. All authors read, critically reviewed, and approved the final manuscript.

### Conflict of Interest Statement

The authors declare that the research was conducted in the absence of any commercial or financial relationships that could be construed as a potential conflict of interest.

## Abbreviations

AmR: Amplex Red
ADIPOQ: Adiponectin
BMAds: Bone marrow adipocytes
CAT: Catalase
CEBPA: CCAAT/enhancer binding protein alpha
Dexa: Dexamethasone
DGAT2: Diacylglycerol O-acyltransferase 2
GLUT4: Glucose transporter type 4
GPX4: Glutathione peroxidase 4
H_2_O_2_: Hydrogen peroxide
HG: High glucose
hBMSCs: Human bone mesenchymal stromal cells
HRP: Horseradish peroxidase
IBMX: 3-isobutyl-1-methylxanthine
ind: Adipogenic inducers
Indo: Indomethacin
KRPG: Krebs-Ringer phosphate buffer
LEP: Leptin
LG: Low glucose
NAC: *N*-acetyl-*L*-cysteine
NOX4: NADPH oxidase 4
PPARG: Peroxisome proliferator activated receptor gamma
PLIN2: Perilipin 2
ROS: Reactive oxygen species
RPL13A: Ribosomal protein L13a
SOD2: Superoxide dismutase 2
TBHP: Tert-butyl hydroperoxide.
