# Supplementary figures and images for: High Glucose Level Impairs Human Mature Bone Marrow Adipocyte Function Through Increased ROS Production
Source: Front Endocrinol (Lausanne). 2019 Sep 10;10:607. doi: 10.3389/fendo.2019.00607 (PMC6746912; doi:10.3389/fendo.2019.00607)

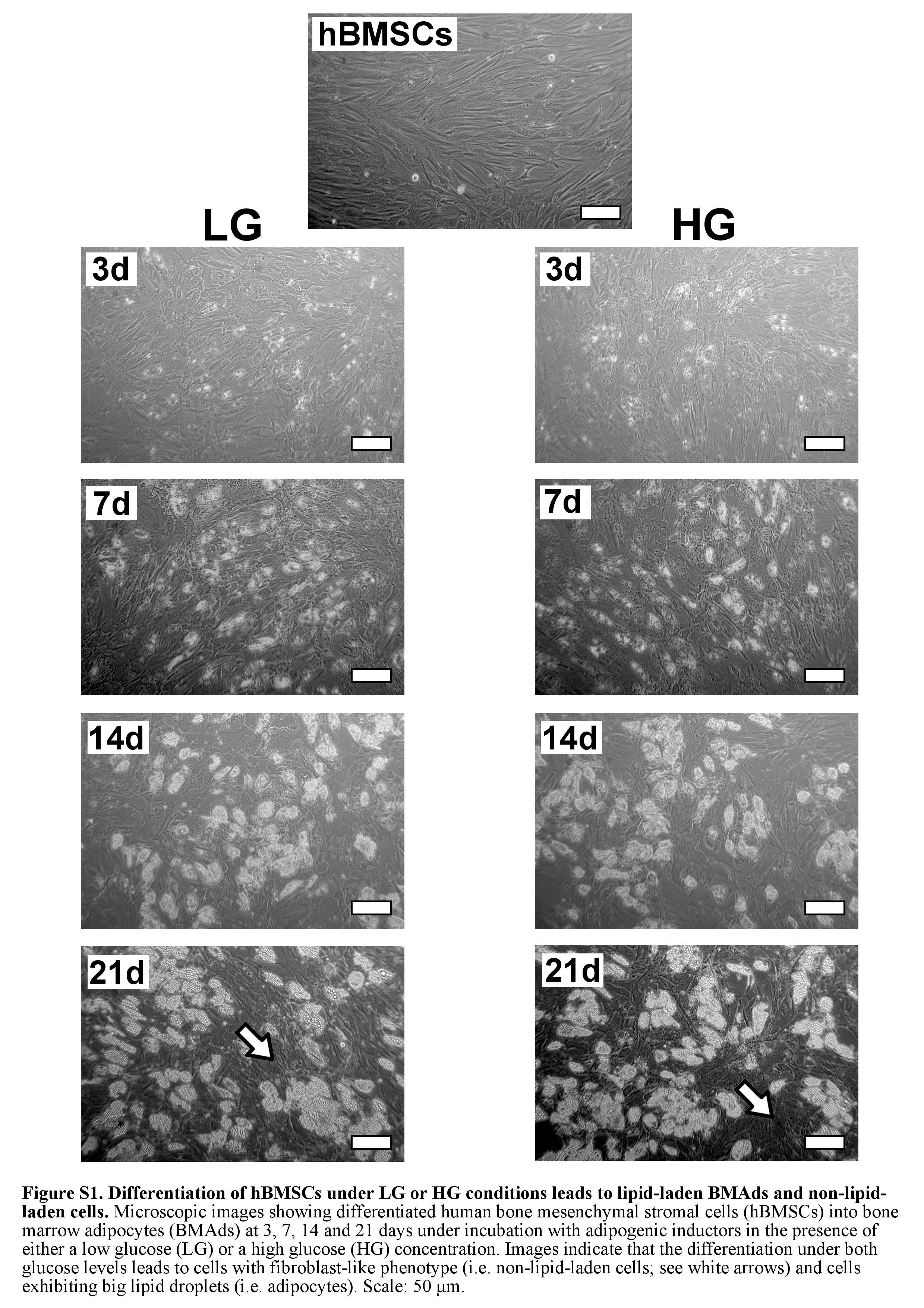

Supplement: Supplementary file 1 [file Image_1.jpg]

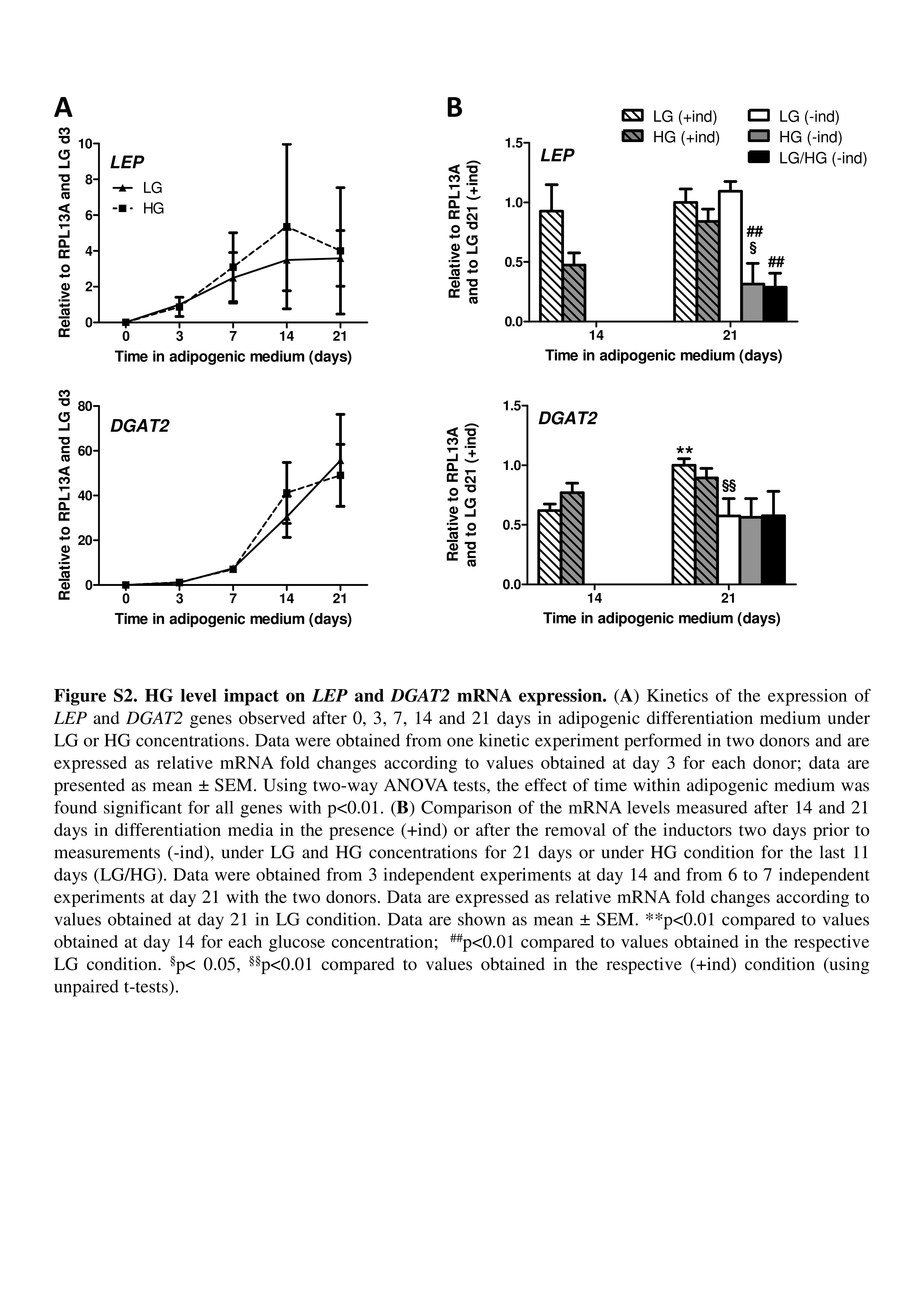

Supplement: Supplementary file 2 [file Image_2.TIF]

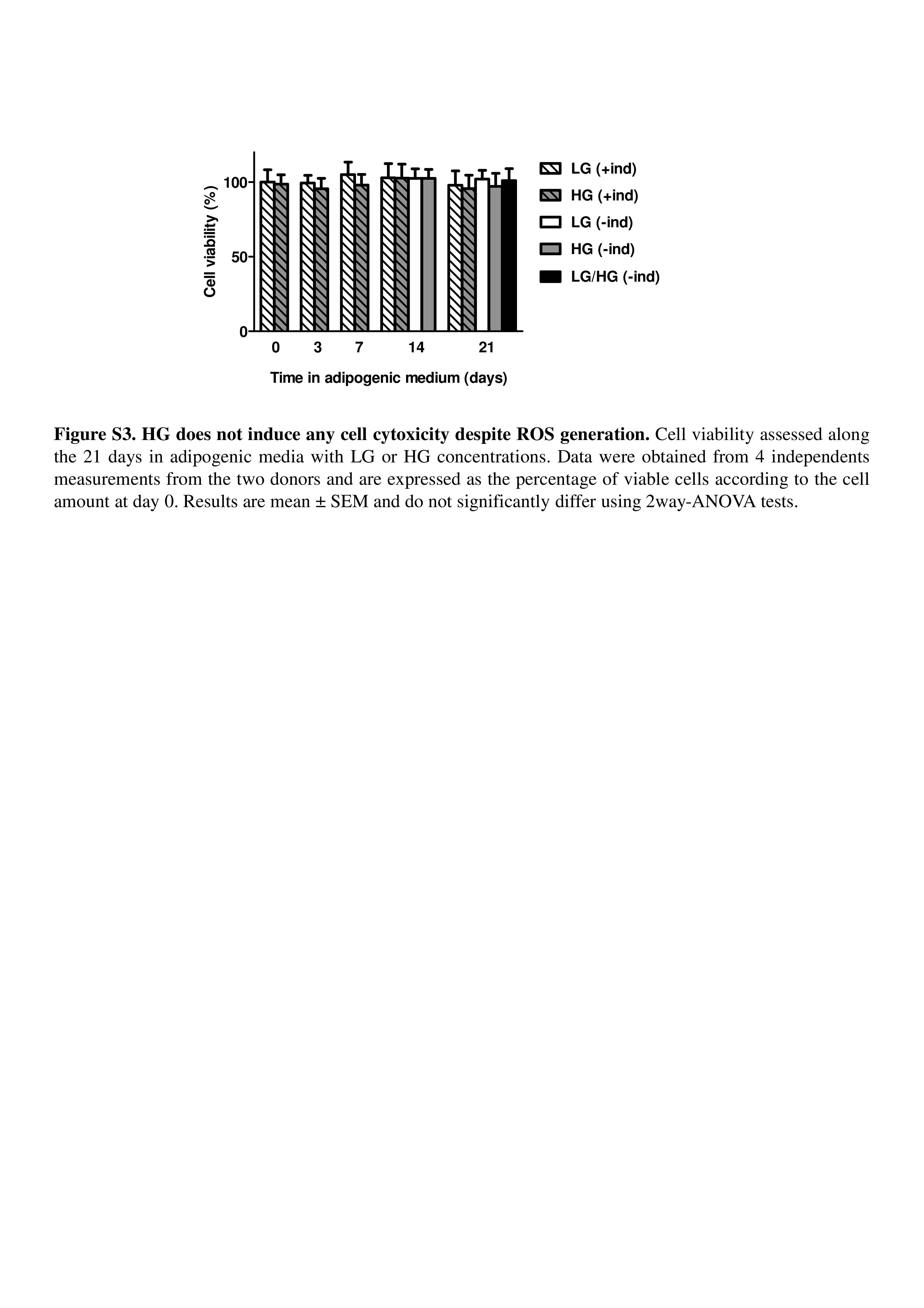

Supplement: Supplementary file 3 [file Image_3.TIF]
