# Supplementary material for: High Glucose Level Impairs Human Mature Bone Marrow Adipocyte Function Through Increased ROS Production
Source: Front Endocrinol (Lausanne). 2019 Sep 10;10:607. doi: 10.3389/fendo.2019.00607 (PMC6746912; doi:10.3389/fendo.2019.00607)
Supplement: Supplementary file 4 [file Image_4.pdf]

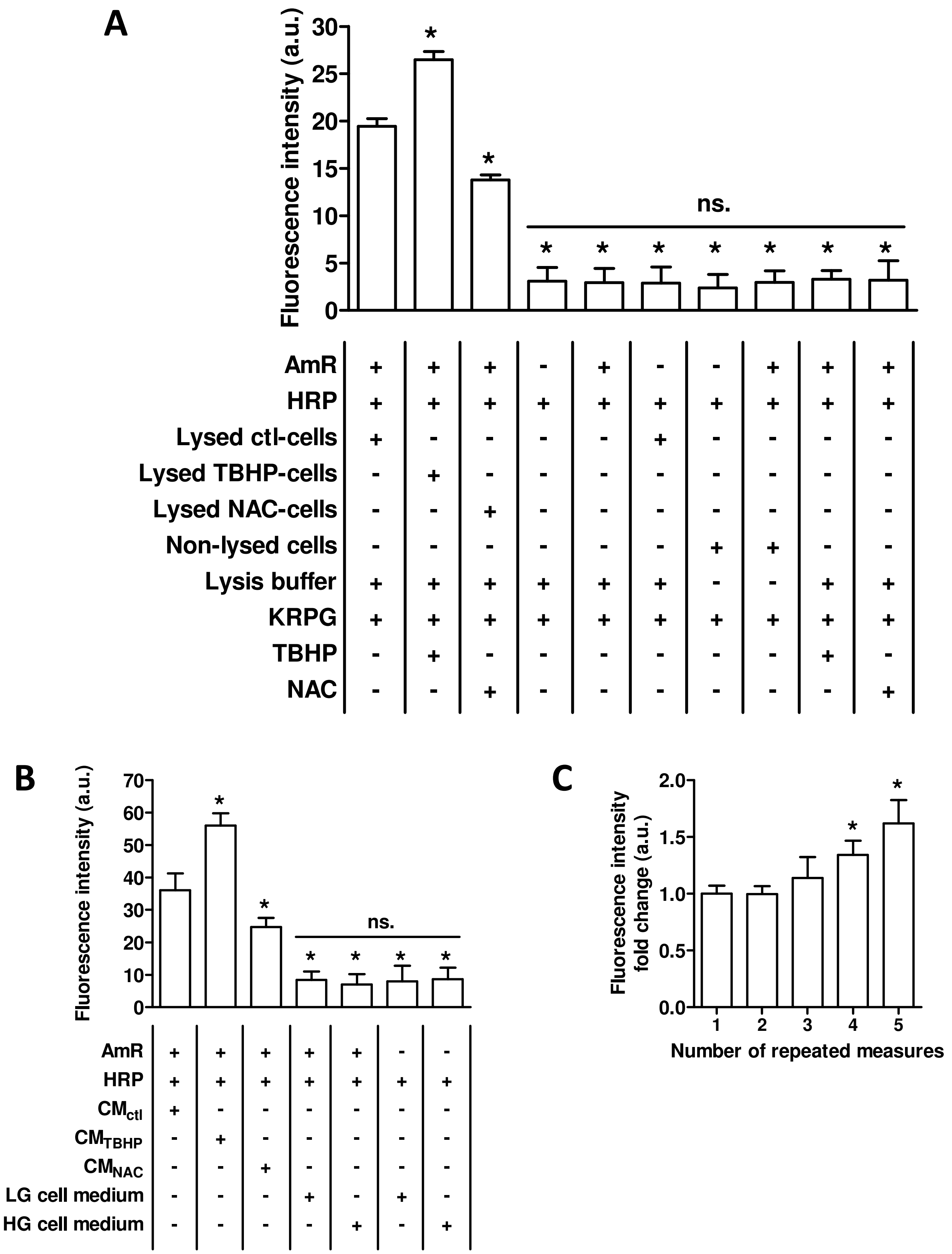

Figure S4. AmR method is specific and sensitive for the detection of intracellular and extracellular ROS levels. (**A**) Assessment of the specificity of AmR to intracellular H_2O_2. Differentiated BMAds from hBMSCs were treated or not with pro- or antioxidant reagents (i.e. 10 µM TBHP or 1 mM NAC, respectively) for one day prior to cell lysis followed by AmR staining. The auto-fluorescence of the probe, the cells, the lysis buffer (LB) and the incubation buffer (KRPG) has been monitored. Data show that the detected fluorescence emission is solely linked to the interaction of the probe with intracellular H_2O_2. *$p<0.05$ in comparison with untreated lysed and AmR-stained untreated cells; ns, non-significant (unpaired t-tests). (**B**) Monitoring of the specificity of AmR to extracellular H_2O_2. Conditioned media (CM) (from

non-lysed BMAds) were loaded with the probe, and fluorescence intensity was compared with the one obtained in CM alone. *$p < 0.05$ in comparison with CM from untreated non-lysed cells loaded with AmR; ns, non-significant. (**C**) Measurement of fluorescence intensities emitted by the probe after several repeated excitations on the same sample. Increased fluorescence emission resulting from a photo-induction of the probe was held only after 4 repeated measures on the same sample, but no photosensitivity of probe was observed under our experimental procedure (i.e. 1 excitation step only). *$p<0.05$ in comparison with 1 excitation step only. All data are presented as mean fluorescence intensity ± SEM (a.u.) from at least three independent experiments.

Results from Figure S4:

We verified the reliability of AmR method (**1-4**) for detecting intracellular H_2O_2 following cell lysis under our experimental conditions (**Figure S4A**). As expected, the fluorescent signal increased in lysed stained cells by comparison with lysed unstained ones. Moreover, the signal either increased or decreased following cell treatment with the pro-oxidant compound tert-butyl hydroperoxide (TBHP) or the antioxidant reagent *N*-acetyl-*L*-cysteine (NAC), respectively. Changes in the fluorescent signal level are solely linked to the reaction between the probe and intracellular H_2O_2 levels. Indeed, neither the lysis buffer nor the loading medium i.e. KRPG, altered the fluorescence property of the probe as the signal remains as negligible as the auto-fluorescence of each solution. Yet, lysing the cells does not induce any change in ROS levels as the detected signal remains similar to the auto-fluorescence emitted by intact cells. Finally, neither TBHP nor NAC directly interact with the probe.

In addition, AmR was found sensitive enough for measuring H_2O_2 extracellularly released by the cells (**Figure S4B**). The probe did not react with any compounds of the cell culture medium since comparable weak fluorescent signals have been detected in culture media with or without AmR supplementation. Furthermore, an increase in the fluorescence signal was detected only when the probe was added to the conditioned medium i.e. culture medium in contact with cells. Finally, changes in the fluorescent signal results from the interaction of the probe with extracellular H_2O_2 only as treatments of the cells with either TBHP or NAC respectively enhanced and reduced the signal intensity. Moreover, changes in the fluorescent signal are not connected to any direct interactions between the probe and the reagents; instead, they are rather linked to the respective pro-oxidant and antioxidant properties of the compounds as no signal changes have been observed in the culture medium supplemented with the probe and each reagent.

Of note, the production of resofurin from AmR has been reported to be a photosensitive reaction (**5**). We therefore examined whether the fluorescence signal of the probe is photo-induced under our experimental procedure (i.e. 1 excitation step). Although the probe exhibited a photosensitivity, it only occurred after four repeated excitation steps as shown by a significant increase in the fluorescence intensity by 1.5 fold (**Figure S4C**). The signal remained unchanged after 2 repeated acquisition on the same AmR-stained sample. It is therefore unlikely that signal changes result from any photo-induction of the probe. Taken together, our data rather ascribe changes in the detected fluorescence intensities following cell staining with AmR to H_2O_2 levels. Accordingly, our results confirm that the AmR method is sensitive and specific enough for ROS detection under our experimental conditions.

References:

1. Zhou M, Diwu Z, Panchuk-Voloshina N, Haugland RP. A stable nonfluorescent derivative of resorufin for the fluorometric determination of trace hydrogen peroxide: applications in detecting the activity of phagocyte NADPH oxidase and other oxidases. *Anal Biochem* (1997) **253**:162–168. doi:10.1006/abio.1997.2391

2. Tormos KV, Anso E, Hamanaka RB, Eisenbart J, Joseph J, Kalyanaraman B, Chandel NS. Mitochondrial complex III ROS regulate adipocyte differentiation. *Cell Metab* (2011) **14**:537–544. doi:10.1016/j.cmet.2011.08.007

3. Rharass T, Gbankoto A, Canal C, Kurşunluoğlu G, Bijoux A, Panáková D, Ribou A-C. Oxidative stress does not play a primary role in the toxicity induced with clinical doses of doxorubicin in myocardial H9c2 cells. *Mol Cell Biochem* (2016) **413**:199–215. doi:10.1007/s11010-016-2653-x

4. Ribou A-C. Synthetic Sensors for Reactive Oxygen Species Detection and Quantification: A Critical Review of Current Methods. *Antioxid Redox Signal* (2016) **25**:520–533. doi:10.1089/ars.2016.6741

5. Zhao B, Summers FA, Mason RP. Photooxidation of Amplex Red to resorufin: implications of exposing the Amplex Red assay to light. *Free Radic Biol Med* (2012) **53**:1080–1087. doi:10.1016/j.freeradbiomed.2012.06.034
